# Chemokine ligand 18 predicts all-cause mortality in patients hospitalized with chest pain of suspected coronary origin

**DOI:** 10.1016/j.ijcrp.2024.200264

**Published:** 2024-03-27

**Authors:** Dennis W.T. Nilsen, Reidun Aarsetoey, Volker Poenitz, Thor Ueland, Pål Aukrust, Annika E. Michelsen, Trygve Brugger-Andersen, Harry Staines, Heidi Grundt

**Affiliations:** a Stavanger University Hospital, Department of Cardiology, Stavanger, Norway; b University of Bergen, Department of Clinical Science, Bergen, Norway; c Department of Clinical Medicine, Thrombosis Research Center, UiT - The Arctic University of Norway, Tromsø, Norway; d University of Oslo, Faculty of Medicine, Oslo, Norway; e Oslo University Hospital, Rikshospitalet, Research Institute of Internal Medicine, Oslo, Norway; f Oslo University Hospital, Rikshospitalet, Section of Clinical Immunology and Infectious Diseases, Oslo, Norway; g Stavanger Heart Center, Stavanger, Norway; h Sigma Statistical Services, Balmullo, Northern Ireland, United Kingdom; i Stavanger University Hospital, Department of Respiratory Medicine, Stavanger, Norway

**Keywords:** Chemokine ligand 18/pulmonary activation regulated chemokine (CCL18/PARC**)**, Biomarkers, Acute coronary syndrome (ACS), Cardiovascular disease (CVD) events, Mortality

## Abstract

**Introduction:**

Chemokines mediate recruitment and activation of leucocytes. Chemokine ligand 18 (CCL18) is mainly expressed by monocytes/macrophages and dendritic cells. It is highly expressed in chronic inflammatory diseases, and locally in atherosclerotic plaques, particularly at sites of reduced stability, and systemically in acute coronary syndrome patients. Reports on its prognostic utility in the latter condition, including myocardial infarction (MI), are scarce.

**Aim:**

To assess the utility of CCL18 as a prognostic marker of recurrent cardiovascular events in patients hospitalized with chest pain of suspected coronary origin.

**Methods:**

The population consisted of 871 consecutive chest-pain patients, of whom 386 were diagnosed with acute myocardial infarction (AMI) based on Troponin-T (TnT) levels >50 ng/L. Stepwise Cox regression models, applying normalized continuous log_e_/SD values, were fitted for the biomarkers with cardiac mortality within 2 years and total mortality within 2 and 7 years as the dependent variables.

**Results:**

Plasma samples from 849 patients were available. By 2 years follow-up, 138 (15.8%) patients had died, of which 86 were cardiac deaths. Univariate analysis showed a positive, significant association between CCL18 and total death [HR 1.55 (95% 1.30–1.83), p < 0.001], and for cardiac death [HR 1.32 (95% 1.06–1.64), p = 0.013]. Associations after adjustment were non-significant. By 7 years follow-up, 332 (38.1%) patients had died. CLL18 was independently associated with all-cause mortality [HR 1.14 (95% CI, 1.01–1.29), p = 0.030], but not with MI (n = 203) or stroke (n = 55).

**Conclusion:**

CCL18 independently predicts long-term all-cause mortality but had no independent prognostic bearing on short-term cardiac death and CVD events.

## 1 Introduction

C–C chemokines are chemotactic cytokines that regulate leucocyte recruitment [1] as well as activation of leucocyte subsets and a range of other cells such as endothelial cells and smooth muscle cells, and are rapidly upregulated at sites of vascular inflammation [1]. Chemokine (C – C motif) ligand 18 (CCL18), also termed pulmonary activation regulated chemokine (PARC), plays a role in homeostasis [2] and is upregulated in various systemic diseases [2,3]. CCL18 is primarily expressed by innate immune cells such as dendritic cells and monocytes/macrophages [3], with among others, effects on cells in adaptive immunity such as T- and B-cells [[3], [4], [5], [6]].

In general, CCL18 is not widely studied in patients with cardiovascular disease, but macrophages within atheroscerotic plaques had increased expression of CCL18, although not associated with lipid loading of the macrophages [7,8]. In relation to ischemic heart disease, CCL18 was found to be significantly elevated in patients with refractory ischemic symptoms versus stabilized patients [9], and high CCL18 levels were associated with fatal events in 609 patients with acute coronary syndrome (ACS) during a follow-up period of 200 days [10]. In patients with stable chest pain, CCL18 correlated with the extent of coronary artery disease (CAD), but did not provide independent prognostic information [11]. The role of CCL18 as a predictor of outcome in CAD patients is, however, far from clear.

The current study was designed to evaluate associations between CCL18 and outcomes in CAD in a chest-pain population with clinically suspected ACS. Two subpopulations, with and without an acute myocardial infarction (AMI), identified by a Troponin-T (TnT) cut-off level of 50 ng/L, were studied separately, to assess the influence of an acute TnT response on the prognostic utility of CCL18 in patients admitted to the hospital with suspected ACS. The patients had a follow-up period of 1 year and 2 and 7 years in relation to pre-defined outcomes, involving all-cause mortality, MI and stroke.

## 2 Materials and methods

### 2.1 Study design and patient population

A total of 871 consecutive patients with chest pain of suspected coronary origin were included in the “Risk Markers in the Acute Coronary Syndrome (RACS)” (ClinicalTrials.gov Identifier: NCT00521976) [12,13] at Stavanger University Hospital, Norway, from November 2002 until September 2003. Exclusion criteria were age <18 years, unwillingness or incapacity to provide informed consent, and prior inclusion. Patients were classified as either AMI or non-AMI patients according to their TnT level at baseline and 6 h after admission, respectively, employing a cut-off value of 50 ng/L. Coronary angiography following admission was performed in 268 patients, and 160 patients were treated with percutaneous coronary intervention (PCI) [12]. The non-AMI group consisted essentially of patients not requiring invasive coronary diagnostics.

Admission levels of CCL18 were available in 849 patients. The flow chart is shown in Supplemental Fig. 1. All-cause mortality, MI, and stroke, respectively, and the combined endpoint of these outcomes were recorded at 1 year, 2 years and 7 years follow-up, whereas recording of cardiac mortality was limited to the first 2 years. The combined cardiac endpoint consisted of cardiac mortality or MI or stroke at 2 years follow-up. The definition of cardiac mortality has previously been described [14]. Clinical follow-up data were obtained from hospital- and public registries and by telephone interview at 30 days, 6, 12, 24 and 84 months, whereas additional information was obtained from general practitioners and nursing homes [12,13]. Information regarding all-cause mortality beyond 2 years was essentially provided from death-registries [13]. Baseline data including information on demographics, smoking habits, clinical history, and general laboratory characteristics were collected at hospital admission. The prognostic utility of CCL18 was compared to that of high sensitivity C-reactive protein (hsCRP) and B-type natriuretic peptide (BNP), previously studied in the same population [13].

Written informed consent was obtained from all patients. The RACS study was approved by the Regional Board of Research Ethics and by the Norwegian Health authorities, and was conducted in accordance with the Helsinki declaration of 1971, as revised in 1983.

### 2.2 Blood sampling procedures and laboratory measurements

Blood was drawn by direct venepuncture of an antecubital vein at hospital admission, applying a minimum of stasis. A second blood sample for measurement of TnT was drawn 6–7 h later. Blood samples were centrifuged for 15 min at 2000 g at 20 °C, and stored at −80 °C. Measurement of TnT, estimated glomerular filtration rate (eGFR), glucose and lipids were performed immediately after centrifugation at the laboratory for Medical Biochemistry at Stavanger University Hospital. Multiple aliquots of ethylene diamine tetraacetic acid (EDTA) plasma, citrated plasma and serum were frozen and stored at −80 °C for later measurements.

### 2.3 CCL18 measurements

CCL18 (ng/mL) (Cat#DY394) was analysed by enzyme-linked immunosorbent assay (ELISA) in duplicate in plasma from 849 patients using antibodies from R&D Systems (Minneapolis, MN, USA), in a 384-well format using a combination of a SELMA (Jena, Germany) pipetting robot and a BioTek (Winooski, VT, USA) dispenser/washer. Absorption was read at 450 nm with wavelength correction set to 540 nm using an ELISA plate reader (BioTek). Intra- and inter-assay coefficients were <10 %. Based on data from our own laboratory, measurements in EDTA and citrated plasma did not differ significantly.

### 2.4 TnT, hsCRP and BNP measurements

TnT was quantiﬁed in serum by a cardiac-speciﬁc second generation TnT ELISA assay from Roche Diagnostics, using a high-afﬁnity cardiac-speciﬁc TnT isoform antibody [12]. The lower limit of detection for the assay was 10 ng/L. An AMI diagnosis was based on a peak TnT >50 ng/L, with a typical rise and fall. hsCRP and BNP were analysed in serum and EDTA-plasma, respectively, as previously described [12].

### 2.5 Statistical analysis

Descriptive statistics are presented as medians with interquartile range (25th – 75th percentile) for continuous data and as numbers and percentages for categorical data. Differences in baseline characteristics were assessed by the Kruskal-Wallis test for continuous data and the Chi-squared test for categorical data. Due to a skewed distribution, CCL18, hsCRP, BNP and eGFR levels were logarithmically transformed to the base-e (log_e_) prior to analysis of continuous values and normalized by dividing by the standard deviation (SD). Pearson's correlation coefficient was calculated to identify a possible relation between the admission level of biomarkers. We did not include a power calculation in this exploratory, single arm, observational study.

Patients were divided into quartiles (Q1-4) according to their CCL18 concentrations. The Kaplan-Meier product limits were used for plotting the times to event and the log-rank test was used to test for the equality of the survival curves. Cox regression models, applying both quartiles and continuous log_e_-transformed values, were fitted for CCL18 with the clinical endpoints as the dependent variables within the defined follow-up periods. Results are shown separately for the univariate analyses and after adjusting for gender, and for age and gender. Furthermore, in a multivariable model, CCL18 was added to the confounders that were selected using a stepwise algorithm. For continuous log_e_-transformed values, we employed HR and 95% CI per SD increase of the biomarker. The hazard ratios presented in the results section are 1-SD on the log scale. Subgroup analyses were performed for patients with or without TnT-release above 50 ng/L at index hospitalization. The interaction between CCL18 and hsCRP was analysed by fitting a Cox model with their quartiles plus their interaction term for all-cause mortality at 7 years follow-up.

Receiver operated characteristics (ROC) analysis was constructed, comparing the area under the curve (AUC) of the individual biomarkers. Using the likelihood ratio test (LRT), a model was built for the assessment of the prognostic utility of log_e_-transformed biomarkers added successively to selected conventional clinical markers, finally testing the prognostic utility of log_e_-transformed CCL18 after addition of the other biomarkers.

Statistics were performed using the statistical package SPSS version 25 (IBM Corp. Armonk, NY). All tests were 2-sided with a significance level of 5% without multiplicity adjustment.

## 3 Results

The recruitment flow-chart is shown in Supplemental Fig. 1. No patient was lost to follow-up. Baseline samples were available in 849 of the 871 included subjects. Median follow-up was 7 years. AMI with TnT >50 ng/L was recorded in 374 patients (44.1%), whereas 475 patients (55.9%) were classified as non-AMI. Baseline characteristics of the total patient population and for quartiles of CCL18 are shown in Table 1, and separate baseline data of AMI and non-AMI patients, are presented in Supplemental Tables 1 and 2, respectively. There was no significant difference in the proportion of males and females with a history of previous MI (χ2(1) = 2.26, p = 0.13). A moderately high proportion of patients in the non-AMI group of patients were treated with cardiovascular drugs; 42.5% were treated with acetylsalicylic acid, 38.7% were taking a statin, 40.2% were on a b-blocker, and 36.2% were taking an angiotensin converting enzyme (ACS) inhibitor or angiotensin 2 receptor blocker (ARB). HsCRP followed the same trend as CCL18 across the quartiles of all patient groups (p < 0.001). An increase in BNP (p < 0.001) and a decrease in eGFR (p < 0.001) from quartile 1 (Q1) to Q4 was noted in the total patient population, with similar trends for the two subgroups (AMI and non-AMI). In the study group as a whole, baseline levels of CCL18 were significantly correlated with CRP (r = 0.21, p < 0.001) and BNP (r = 0.24, p < 0.001). However, the interaction terms between the quartiles of CCL18 and each of CRP and BNP was not significant (p = 0.31 and p = 0.51 respectively) in the Cox models for all-cause death at 7 years.

**Table 1:** Baseline characteristics in 871 chest-pain patients with suspected coronary origin, arranged in quartiles (Q) of CCL18, measured in ng/mL. Blood samples for measurement of CCL18 were available in 849 patients.

	Quartile1	Quartile2	Quartile3	Quartile4	p_value	total
	(n = 212)	(n = 212)	(n = 213)	(n = 212)		(n = 849)
CCL18 (ng/mL)	74.9 (63.3–83.8)	105.3 (98.0–112.5)	139.5 (129.1–149.2)	190.8 (172.6–223.6)	<0.001 [2]	120.9 (92.0–160.0)
Age (years)	65.8 (52.0–75.5)	69.1 (57.0–79.9)	74.6 (62.9–82.5)	76.5 (69.1–83.7)	<0.001 [2]	72.6 (59.0–81.1)
Risk markers at baseline;						
Male gender	122 (57.55)	129 (60.85)	134 (62.91)	135 (63.68)	0.570 [1]	520 (61.25)
hs-CRP mg/L	2.6 (1.4–8.3)	3.4 (1.4–9.6)	4.4 (2.0–13.6)	7.2 (2.3–21.0)	<0.001 [2]	4.0 (1.7–13.4)
BNP pg/mL	68.5 (28.0–235.0)	69.5 (26.5–215.5)	112.5 (37.0–320.0)	196.0 (60.0–600.0)	<0.001 [2]	97.5 (34.0–310.5)
eGFR ml/min/1.73m2	69.8 (58.4–82.4)	64.2 (52.2–75.0)	63.2 (47.1–73.0)	54.8 (38.0–68.7)	<0.001 [2]	63.3 (48.7–75.3)
Total cholesterol (mmol/L)	5.4 (4.6–6.3)	5.2 (4.3–6.1)	5.2 (4.2–5.9)	4.9 (4.0–5.8)	0.001 [2]	5.2 (4.3–6.0)
TnT release (>50 ng/L)	94 (44.34)	105 (49.53)	113 (53.05)	146 (68.87)	<0.001 [1]	458 (53.95)
Smoking					0.209 [1]	
Never smoked	73 (34.43)	72 (33.96)	86 (40.38)	87 (41.04)		318 (37.46)
Current smoker	64 (30.19)	55 (25.94)	57 (26.76)	43 (20.28)		219 (25.80)
Ex smoker	75 (35.38)	85 (40.09)	70 (32.86)	82 (38.68)		312 (36.75)
Hypertension	84 (39.62)	84 (39.62)	91 (42.72)	98 (46.23)	0.461 [1]	357 (42.05)
Diabetes mellitus type I	5 (2.36)	1 (0.47)	0 (0.00)	2 (0.94)	0.069 [1]	8 (0.94)
Diabetes mellitus type II	17 (8.02)	24 (11.32)	33 (15.49)	35 (16.51)	0.033 [1]	109 (12.84)
Total chol. >6.5 mmol/L	44 (20.75)	36 (16.98)	32 (15.02)	19 (8.96)	0.008 [1]	131 (15.43)
*History of heart disease:*						
Angina pectoris	81 (38.21)	87 (41.04)	100 (46.95)	108 (50.94)	0.037 [1]	376 (44.29)
Myocardial infarction	66 (31.13)	62 (29.25)	68 (31.92)	86 (40.57)	0.064 [1]	282 (33.22)
Previous CABG	27 (12.74)	20 (9.43)	20 (9.39)	20 (9.43)	0.593 [1]	87 (10.25)
Previous PCI	21 (9.91)	21 (9.91)	24 (11.27)	21 (9.91)	0.956 [1]	87 (10.25)
Heart failure	35 (16.51)	43 (20.28)	61 (28.64)	89 (41.98)	<0.001 [1]	228 (26.86)
*Prehospital treatment:*						
ACEI/ARB	61 (28.77)	66 (31.13)	74 (34.74)	87 (41.04)	0.044 [1]	288 (33.92)
Beta-blocker	75 (35.38)	72 (33.96)	72 (33.80)	87 (41.04)	0.362 [1]	306 (36.04)
Statins	78 (36.79)	76 (35.85)	73 (34.27)	66 (31.13)	0.630 [1]	293 (34.51)
Aspirin	87 (41.04)	83 (39.15)	79 (37.09)	73 (34.43)	0.538 [1]	322 (37.93)

Data are presented as median (interquartile range) or numbers (%). Abbreviations: hs-CRP, high-sensitivity C-reactive protein; BNP, B-type.

natriuretic peptide; eGFR, estimated glomerular filtration rate; TnT, troponin-T; Total chol., total cholesterol; CABG, coronary artery bypass grafting; PCI, percutaneous coronary intervention. ACEI, Angiotensin-converting-enzyme inhibitor; ARB, Angiotensin receptor blocker. *For the diagnosis of an acute myocardial infarction, we applied a cut-off value for TnT of 50 ng/L and the lowest detectable value was 10 ng/L [1] Chi-squared test. [2] Kruskal-Wallis test.

The results of the univariate and adjusted analyses are presented for 1 year, 2 years and 7 years follow-up and includes 1) All patients, 2) AMI patients and 3) Non-AMI patients, employing separate and combined endpoints. In addition to age and gender, other baseline variables were also included in the multivariable model, and those that appeared as significant confounders were adjusted for. Supplemental Table 3 shows significant confounders related to the prognostic utility of CCL18 for selected endpoints in the total population and the two subgroups.

### 3.1 1-year follow-up

In the univariate analysis for the **total patient population** at one year, CCL18 was strongly associated with all-cause mortality (p < 0.001), cardiac death (p = 0.005), MI (p = 0.012) and with stroke (p = 0.015). The results were significant after adjusting for gender. However, after adjusting for age and gender, and possible confounders in the multivariable model, no association with any of the endpoints was found (Table 2A).

**Table 2:** **A & B.** Univariate and multivariable Cox regression model applying continuous log_e_-transformed values of baseline CCL18 values during 1 and 2 years follow-up, respectively, in 871 patients with a suspected acute coronary syndrome. (22 missing values in the univariate and age & gender adjusted and 51 missing values in the multivariable analysis).

A.	All-cause mortality 1 year N = 101 (11.9%) M = 98 (12.0%)		Cardiac death 1 year N = 66 (7.8%) M = 64 (7.8%)		MI 1 year N = 90 (10.6%) M = 87 (10.6%)		Stroke 1 year N = 15 (1.8%) M = 14 (1.7%)	
	HR (95% CI)	P-value	HR (95% CI)	P-value	HR (95% CI)	P-value	HR (95% CI)	P-value
**Univariate**	1.57 (1.30–1.92)	<0.001	1.42 (1.11–1.81)	0.005	1.31 (1.06–1.61)	0.012	1.90 (1.14–3.16)	0.015
**Adjusted for gender**	1.60 (1.31–1.95)	<0.001	1.44 (1.13–1.83)	0.004	1.32 (1.07–1.62)	0.010	1.91 (1.14–3.21)	0.013
**Adjusted for age and gender**	1.22 (0.99–1.50)	0.062	1.07 (0.82–1.38)	0.62	1.11 (0.89–1.39)	0.36	1.34 (0.78–2.31)	0.29
**Multivariable**	1.06 (0.86–1.30)	0.60	0.89 (0.69–1.15)	0.38	0.93 (0.75–1.16)	0.51	1.36 (0.77–2.38)	0.29

**B.**	**All-cause mortality** **2 years** N = 135 (15.9%) M = 129 (15.7%)		**Cardiac death** **2 years** N = 84 (9.9%) M = 80 (9.8%)		**MI** **2 years** N = 151 (17.8%) M = 146 (17.8%)		**Stroke** **2 years** N = 27 (3.2%) M = 26 (3.2%)	
	**HR (95% CI)**	**P-value**	**HR (95% CI)**	**P-value**	**HR (95% CI)**	**P-value**	**HR (95% CI)**	**P-value**
**Univariate**	1.54 (1.30–1.83)	0.087	1.32 (1.06–1.64)	0.013	1.30 (1.10–1.53)	0.002	1.79 (1.22–2.64)	0.003
**Adjusted for gender**	1.57 (1.32–1.86)	<0.001	1.34 (1.08–1.67)	0.008	1.31 (1.11–1.54)	0.001	1.81 (1.23–2.67)	0.003
**Adjusted for age and gender**	1.18 (0.98–1.41)	0.075	0.97 (0.77–1.22)	0.80	1.06 (0.89–1.26)	0.54	1.40 (0.93–2.10)	0.11
**Multivariable**	1.06 (0.88–1.27)	0.54	0.80 (0.64–1.01)	0.056	0.89 (0.75–1.06)	0.19	1.42 (0.94–2.14)	0.10

AbbreviationsN= Number of events in the univariate analysis. M = Number of events in the multivariable analysis. MI = Myocardial infarction. Stroke = Cerebral stroke. HR, Hazard Ratio; 95% CI, 95% confidence interval.

For **AMI patients,** univariate analysis revealed no association between CCL18 and all-cause mortality, cardiac death or MI, whereas the results for stroke were inconclusive due to the low number of patients experiencing this condition, and no associations were noted after adjusting for age and gender, and confounders in the multivariable model (Supplemental Table 4A).

In the **non-AMI group,** a significant association (p < 0.001) was noted between CCL18 and all-cause mortality in the univariate analysis and after adjusting for age and gender (p = 0.004), and remained significant (p = 0.029) in the multivariable analysis (Supplemental Table 6A). Results for other endpoints were non-significant after multivariable adjustment.

### 3.2 2-years follow-up

In the univariate analysis for the **total patient population** at two years, the association of CCL18 with all-cause mortality was weaker (p = 0.087) but remained significant for cardiac death (p = 0.013), MI (p = 0.002) and stroke (p = 0.003). The results were also significant after adjusting for gender. However, after adjustment for age and gender, and for multiple confounders, the associations were no longer significant. These data are shown in Table 2B.

For the **AMI patients**, univariate analysis showed a significant association between CCL18 and all-cause mortality (p = 0.042), but not for the other events (Supplemental Table 4B), and no significance was obtained after adjustment for age and gender, and for multiple confounders, respectively.

In **patients with non-AMI**, however, univariate analysis showed a positive association between CCL18 and all-cause mortality (p < 0.001), cardiac death (p = 0.024), MI (p = 0.009), and stroke (p = 0.019). The results were significant after adjusting for gender. After adjusting for age and gender, the relationship remained significant for all-cause mortality (p = 0.020), but not for cardiac death and the non-fatal endpoints, and after adjusting for multiple confounders no significant associations were obtained. (Supplemental Table 6B).

### 3.3 7-years follow-up

An independent positive association between CCL18 and all-cause mortality was noted by the fourth year of follow-up [HR 1.17 (95%CI 1.01–1.36), p = 0.041] (Supplemental Table 7). By 7 years of follow-up, the positive association between CCL18 and all-cause mortality **in the patients as a whole** (Table 3) remained highly significant in the univariate analysis (p < 0.001), and the association with MI was also significant (p < 0.001), whereas the association with stroke was non-significant (p = 0.18). As further depicted in Table 3 the association with all-cause mortality, but not for other outcomes at 7 years (MI or stroke), was maintained after adjusting for age and gender (p = 0.003), and for multiple confounders (p = 0.030), respectively.

**Table 3:** Univariate and multivariable Cox regression model applying continuous log_e_-transformed values of baseline CCL18 values during median 7 years follow-up in 871 patients with a suspected acute coronary syndrome. (22 missing values in the univariate and age & gender adjusted and 51 missing values in the multivariable analysis).

	All-cause mortality 7 years N = 327 (38.5%) M = 317 (38.7%)		MI 7 years N = 199 (23.4%) M = 193 (23.5%)		Stroke 7 years N = 52 (6.1%) M = 51 (6.2%)	
	**HR (95% CI)**	**P-value**	**HR (95% CI)**	**P-value**	**HR (95% CI)**	**P-value**
**Univariate**	1.58 (1.41–1.77)	<0.001	1.28 (1.11–1.48)	0.001	1.21 (0.91–1.60)	0.18
**Adjusted for gender**	1.60 (1.43–1.79)	<0.001	1.28 (1.11–1.48)	<0.001	1.23 (0.93–1.64)	0.14
**Adjusted for age and gender**	1.20 (1.06–1.35)	0.003	1.04 (0.90–1.22)	0.58	0.94 (0.70–1.27)	0.71
**Multivariable**	1.14 (1.02–1.29)	0.030	0.93 (0.80–1.08)	0.35	0.95 (0.71–1.27)	0.72

	All-cause mortality or MI 7 years N = 397 (46.8%) M = 385 (47.0%)		All-cause mortality or MI or Stroke 7years N = 413 (48.6%) M = 400 (48.8%)	
	**HR (95% CI)**	**P-value**	**HR (95% CI)**	**P-value**
**Univariate**	1.39 (1.25–1.54)	<0.001	1.38 (1.24–1.52)	<0.001
**Adjusted for gender**	1.40 (1.26–1.55)	<0.001	1.38 (1.25–1.53)	<0.001
**Adjusted for age and gender**	1.09 (0.98–1.22)	0.10	1.08 (0.98–1.20)	0.14
**Multivariable**	1.02 (0.91–1.14)	0.75	1.01 (0.91–1.13)	0.79

Abbreviations: N= Number of events in the univariate analysis. M = Number of events in the multivariable analysis. MI = Myocardial infarction. Stroke = Cerebral stroke. HR, Hazard Ratio; 95% CI, 95% confidence interval.

**Fig. 1:**
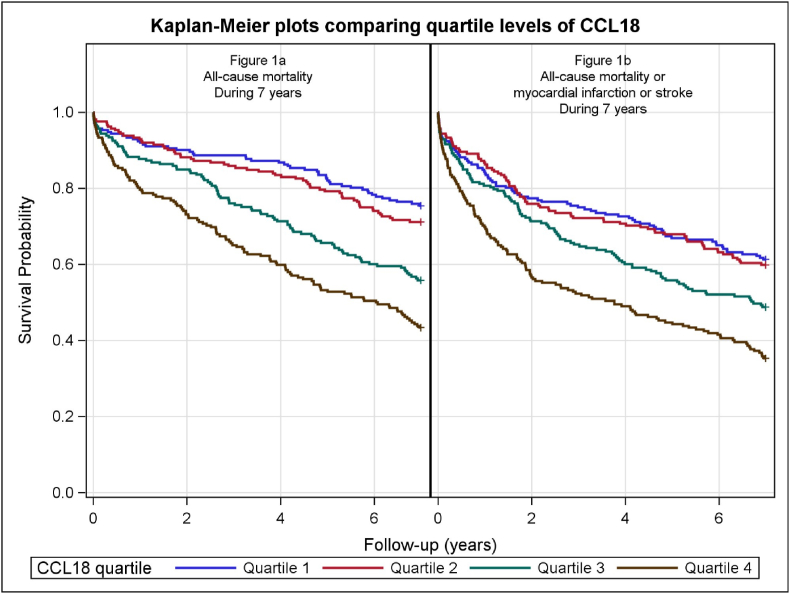
a–b.

The Kaplan-Meier curves comparing quartile levels of CCL18 for all-cause mortality and the composite endpoint (all-cause mortality or MI or stroke) at 7 years follow-up, respectively, are depicted in Fig. 1a–b, each with a log-rank test of p < 0.001.

In the **AMI patients**, CCL18 was found to be significantly associated with all-cause mortality (p < 0.001) in univariate analysis, and after adjusting for gender, but not after adjustment for age and gender, and for multiple confounders. No significant associations were found with MI or stroke (Supplemental Table 5).

**Patients with non-AMI** (Supplemental Table 8) showed a positive association between CCL18 and all-cause mortality in the univariate analysis (p < 0.001), and after adjusting for gender (p < 0.001), age and gender (p = 0.003) and for multiple confounders (p = 0.030), respectively. Except for a significant association with MI in univariate analysis (p = 0.002), no other significant associations with outcome were revealed.

Based on the diagnostic grouping of non-AMI patients in Supplemental Table 9, no significant interaction effect was found between index diagnosis and CCL18 quartile (p = 0.45).

### 3.4 Analysis of cardiac mortality and cardiovascular combined endpoint

During the first 2 years, cardiac mortality was recorded in addition to all-cause mortality. In the univariate analysis at 1 and 2 years, the association of CCL18 and cardiac mortality **in the total population** was statistically significant with HRs of 1.42 (p = 0.005) and 1.32 (p = 0.013), respectively. The results remained significant after adjusting for gender but were no longer significant after adjusting for age and gender, and for multiple confounders, respectively (Table 2. A & B). Similar univariate results at 1 and 2 years were obtained from the analysis of the combined cardiovascular endpoint consisting of cardiac mortality or MI or stroke, with a p-value of <0.001 at both follow-up periods. However, no significant results were observed in the multivariable analysis (Supplemental Table 7)

Table 4 A shows the univariate and adjusted results at 2 years follow-up of the association between CCL18 and cardiac mortality, and between CCL18 and the combined endpoints consisting of cardiac mortality or MI or stroke, for the AMI and non-AMI group of patients, respectively. No significant associations were obtained in the AMI group of patients, in the univariate analysis, or after adjusting for gender, for age and gender or for multiple confounders.

**Table 4:** **A:** Univariate and multivariable Cox regression model applying continuous log_e_-transformed values of baseline CCL18 values during 2 years follow-up in 386 patients with an acute myocardial infarction (AMI) (TnT >50 ng/L), with 12 missing values in the univariate and age & gender adjusted analyses and 25 missing values in the multivariable analysis, and in 485 patients with non-AMI (TnT ≤50 ng/L), with 10 missing values in the univariate and age & gender adjusted analyses and 26 missing values in the multivariable analysis. **B:** Univariate and multivariable Cox regression model applying continuous log_e_-transformed values of baseline CCL18 values during 7 years follow-up in 386 patients with AMI and 485 patients with non-AMI, respectively.

A	Troponin-T > 50 ng/L AMI 2 years follow-up				Troponin-T ≤ 50 ng/L Non-AMI 2 years follow-up			
	Cardiac mortality N = 55 (14.7%) M = 53 (14.7%)		Cardiac mortality or MI or stroke N = 128 (34.2%) M = 125 (34.2%)		Cardiac mortality N = 29 (6.1%) M = 27 (5.9%)		Cardiac mortality or MI or stroke N = 89 (18.7%) M = 85 (18.5%)	
Statistical analysis	HR (95% CI)	P-value	HR (95% CI)	P-value	HR (95% CI)	P-value	HR (95% CI)	P-value
**Univariate**	1.14 (0.87–1.49)	0.34	1.16 (0.97–1.38)	0.11	1.56 (1.06–2.29)	0.024	1.49 (1.20–1.86)	<0.001
**Adjusted for gender**	1.18 (0.91–1.55)	0.21	1.16 (0.97–1.39)	0.098	1.55 (1.06–2.26)	0.024	1.47 (1.18–1.83)	<0.001
**Adjusted for age and gender**	0.89 (0.67–1.19)	0.43	0.96 (0.79–1.15)	0.64	1.07 (0.72–1.58)	0.74	1.10 (0.88–1.38)	0.39
**Multivariable**	0.88 (0.63–1.21)	0.43	0.87 (0.72–1.06)	0.16	*Did not converge		0.95 (0.76–1.18)	0.62

**B**	**7 years follow-up**				**7 years follow-up**			
	**All-cause mortality** N = 150 (40.1%) M = 147 (40.7%)		**All-cause mortality or MI or stroke** N = 199 (53.2%) M = 194 (53.7%)		**All-cause mortality** N = 177 (37.3%) M = 170 (37.0%)		**All-cause mortality or MI or stroke** N = 214 (45.1%) M = 206 (44.9%)	
**Statistical analysis**	**HR (95% CI)**	**P-value**	**HR (95% CI)**	**P-value**	**HR (95% CI)**	**P-value**	**HR (95% CI)**	**P-value**
**Univariate**	1.42 (1.21–1.67)	<0.001	1.19 (1.03–1.38)	0.017	1.77 (1.51–2.08)	<0.001	1.56 (1.35–1.80)	<0.001
**Adjusted for gender**	1.44 (1.23–1.70)	<0.001	1.21 (1.05–1.39)	0.010	1.77 (1.51–2.08)	<0.001	1.56 (1.35–1.80)	<0.001
**Adjusted for age and gender**	1.44 (1.23–1.70)	<0.001	1.00 (0.86–1.16)	0.97	1.31 (1.10–1.56)	0.003	1.18 (1.01–1.38)	0.041
**Multivariable**	1.12 (0.94–1.33)	0.23	0.95 (0.81–1.11)	0.52	1.20 (1.02–1.42)	0.030	1.09 (0.93–1.26)	0.28

In the **non-AMI patients**, significant associations between CCL18 and cardiac mortality (p = 0.024), and the combined cardiovascular endpoint (p < 0.001) were noted in the univariate analysis at 2 years follow-up The results remained significant after adjusting for gender but were no longer significant after the other adjustments.

Table 4B shows the univariate and the adjusted associations between CCL18 and the combined endpoint of all-cause mortality or MI or stroke at 7 years follow-up in AMI and non-AMI patients, respectively. The associations were significant for both subgroups in the univariate analysis, with p-values of 0.017 and <0.001, respectively, maintained in the non-AMI group of patients after adjusting for age and gender (p = 0.041), whereas the other adjustments were statistically non-significant.

### 3.5 Receiver operated characteristic (ROC) analysis

**Fig. 2:**
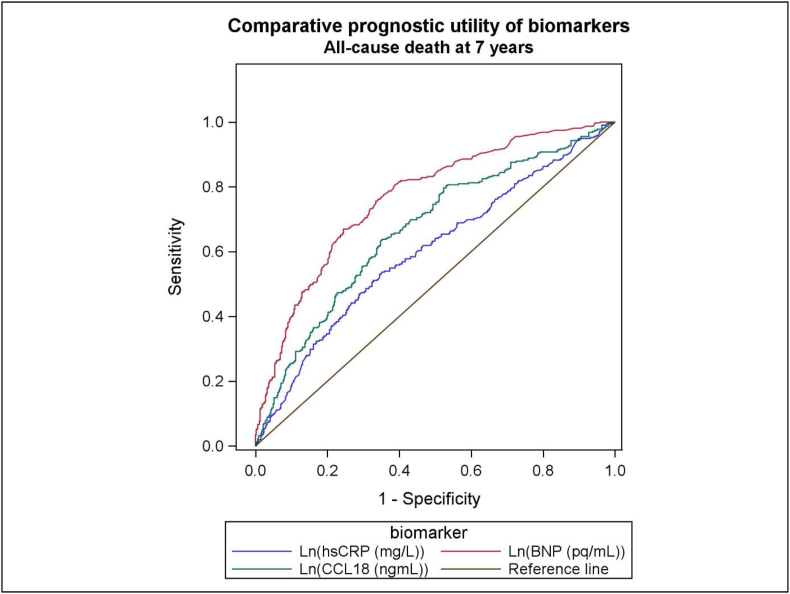
Receiver operated characteristic (ROC) curves depicting the relationship between CCL18, hsCRP and BNP, respectively, and all-cause mortality in the total population at 7-years follow-up.

The ROC curves in Fig. 2 for the total population relate to all-cause mortality at 7-years follow-up and shows the log_e_-transformed AUC values, as follows: CCL18 (0.66, p < 0.001), hsCRP (0.61, p < 0.001) and BNP (0.77, p < 0001), respectively.

For total mortality at 7 years follow-up in the non-AMI subset of patients, the area under the ROC curves for log_e_-transformed AUC values of CCL18 stratified for index diagnosis was 0.59 for unstable angina and 0.66 for non-ACS, respectively.

### 3.6 Effect of CCL18 in addition to conventional risk factors and existing biomarkers

The likelihood ratio test was used to assess the effect of modelling conventional risk factors, existing biomarkers and CCL18 for all-cause mortality at seven years. Adding log_e_ hsCRP and TnT to selected conventional risk factors produced a significant better model for prediction of all-cause mortality at 7 years follow-up [p < 0.001, mainly driven by TnT (≤10 vs > 10 ng/L). The latter model was improved by adding BNP (p < 0.001). Finally, by adding log_e_ CCL18 on top of conventional risk factors, TnT, hsCRP and BNP, a significant additional improvement (p = 0.033) was recorded.

In the subset of patients with coronary angiography (Supplemental Fig. 2), the distributions of CCL18 were similar across the number of diseased vessels, and there was no evidence of CCL18 depending on the extent of affected coronary vessels, p = 0.96.

## 4 Discussion

Data on CCL18 levels in ACS are scarce. In this study with an observational period of seven years, adjusting for gender alone made very little difference to the statistically significant CCL18 HR results in the univariate analysis, whereas adding age as well cancelled out most of the significant univariate results. However, after adjusting for multiple confounders, CCL18 remained associated with all-cause mortality, first recorded as statistically significant at four years follow-up in the total population, reflecting a significant association in the non-AMI population, not observed in AMI patients.

In non-AMI patients, CCL18 was found to be an independent predictor of all-cause mortality during the first year and at 7 years follow-up. As judged by the area under the ROC curve, CCL18 in this subgroup added significant prognostic information beyond that of the index diagnosis, especially in subjects not presenting with unstable angina.

Employing LRT, the model was improved for the total population in relation to all-cause mortality by adding CCL18 on top of conventional risk factors, TnT, hsCRP and BNP.

Cardiac mortality was followed for up to two years. During this period no association was found in the adjusted analysis between CCL18 and cardiac death or the combined cardiovascular endpoint. Furthermore, no independent association was found between CCL18 and MI or stroke during the entire follow-up period.

Our findings for the first time show an independent association of CCL18 and long-term total mortality in patients admitted to hospital with suspected ACS. However, our findings do not support an independent association between CCL18 and future cardiovascular events, such as MI, stroke and cardiac mortality, and the independent association with total mortality was restricted to the non-AMI patients. Our findings are in some degree consistent with a previous report investigating the association between CCL18 and cardiovascular events in 712 stable chest-pain patients undergoing CT cardiac angiography [11]. In that study with a mean follow-up of 26 months, 51 events, counting all-cause mortality, ACS or coronary revascularizations, were recorded. CCL18 was significantly associated with coronary calcification, but did not provide prognostic information with regard to future cardiac events [11]. That study, however, did not examine long-term mortality. Our findings are also to some degree in agreement with those of another study counting 609 patients, in whom CCL18 was found to be independently associated with short-term (200 days) fatal events in patients with ACS, but not with non-fatal events [10]. However, the latter study did not account for cardiac deaths among patients suffering a fatal event, and again had no data on long-term total mortality.

In our study, the coronary anatomy of the non-AMI population was only characterized in a small subset of patients undergoing coronary angiography, in which CCL18 was not found to be related to obstructive vessel engagement. The strength of our study would be a vast number of recorded cardiovascular endpoints during long-term follow-up, enabling us to investigate the relation between CCL18 levels and clinical cardiovascular deterioration. Also, not only the AMI population, but also a large number of non-AMI patients present with clinically established coronary heart disease. Accordingly, it has previously been shown that patients admitted to hospital with acute chest pain are at risk for future events, partly independent of the origin of the pain [15], and the present study may further support such a notion.

The reason for the link between CCL18 and all-cause mortality is at present not clear, but high expression of CCL18 has been associated with several medical conditions involving cancer, immunological and inflammatory diseases, and Gaucher disease [16]. Inflammation *per se* is an independent risk factor for total mortality [17] and that should also include raised levels of CCL18. Moreover, although CCL18 was not an independent risk factor of cardiovascular outcome, cardiac death was a major contributor to total mortality, accounting for 62% of deaths at 2 years follow-up in the total population, 74% among AMI patients, and 48% in the non-AMI group, implying the need for larger studies to make any firm conclusion. Further studies are also needed to clarify which mechanisms of disease entities contribute to the association of CCL18 and all-cause mortality in non-AMI patients.

## 5 Limitations

The RACS registry was established in 2002, in a modern clinical setting with available on-site invasive treatment, when required. Patients were sorted by a second-generation TnT into a group with and without AMI, respectively. A more sensitive assay would have picked up some patients with minor myocardial injury between 50 ng/L in the assay used and 14 ng/L in the high-sensitivity assay introduced some years later in 2010 [18]. However, this would not have affected the adjusted association between CCL18 and cardiac endpoints, which was non-significant in the total material, and remained so in the two separate subgroups.

Only approximately one third of the included population had coronary angiography performed during hospitalization. The use of medication was recorded only at admission but reflects a high frequency of patients with established CHD in the non-AMI subgroup. However, aspirin and a statin would routinely have been offered before discharge to all patients with suspected CHD. Furthermore, all patients presented with anginal pain at admission, underscoring the presence of CHD in a majority of subjects. Blood sampling was limited to one draw at hospital admission. All subjects were recruited from a Norwegian population, and our results may not necessarily be generalizable. Finally, the lack of recorded cardiac mortality at seven years follow-up limits the conclusion of CCL18 as a marker for long-term cardiovascular events.

In conclusion, CLL18 independently predicted long-term all-cause mortality in patients admitted with acute chest-pain of suspected coronary origin. Its utility as an independent predictor of all-cause mortality was limited to the non-AMI cohort. The lack of associations with cardiac events (i.e., cardiac mortality and MI) suggests that CCL18 is not a good marker of atherothrombotic events.

## Statement of Ethics

The RACS study was approved by the Regional Board of Research Ethics (approval no. 118.02, 2010/1074-3) and by the Norwegian Health authorities. Written informed consent was obtained from participants to participate for 2-years and for long-term follow-up. The study was conducted in accordance with the Helsinki declaration of 1971, as revised in 1983.

## Funding

This work was supported by the Western Norway Regional Health Authority and 10.13039/501100010738Stavanger University Hospital, Norway, throughout the study, and partly by Axis-Shield, Dundee, UK, during the first 2 years of the study.

## Data availability Statement

The datasets analysed during the current study are not publicly available due to Norwegian legislation regarding general data protection regulation but are available from the corresponding author, on reasonable request.

## CRediT authorship contribution statement

**Dennis W.T. Nilsen:** Conceptualization, Funding acquisition, Investigation, Methodology, Project administration, Resources, Supervision, Validation, Writing – original draft, Writing – review & editing. **Reidun Aarsetoey:** Investigation, Validation, Writing – review & editing. **Volker Poenitz:** Investigation, Supervision, Validation, Writing – review & editing. **Thor Ueland:** Investigation, Methodology, Validation, Writing – review & editing, Supervision. **Pål Aukrust:** Investigation, Methodology, Supervision, Validation, Writing – review & editing. **Annika E. Michelsen:** Methodology, Validation, Writing – review & editing, Investigation. **Trygve Brugger-Andersen:** Investigation, Supervision, Validation, Writing – review & editing. **Harry Staines:** Data curation, Formal analysis, Methodology, Validation, Writing – review & editing. **Heidi Grundt:** Investigation, Supervision, Validation, Writing – review & editing.

## Declaration of Competing interest

The authors have no conflicts of interest to declare.

All authors take responsibility for all aspects of the reliability and freedom from bias of the data presented and their discussed interpretation.

## References

[1] Charo I.F., Taubman M.B. (2004). Chemokines in the pathogenesis of vascular disease. Circ. Res..

[2] Schutyser E., Richmond A., van Damme J. (2005). Involvement of CC chemokine ligand (CCL18) in normal and pathological processes. J. Leukoc. Biol..

[3] Weber C., Schober A., Zernecke A. (2004). Key regulators of mononuclear cell recruitment in aterosclerotic vascular disease. Arterioscler. Thromb. Vasc. Biol..

[4] Adema G.J., Hartgers F., Verstraten R., de Vries E., Marland G., Menon S., Foster J., Xu Y., Nooyen P., McClanahan T., Bacon K.B., Figdor C.G. (1997). A dendritic-cell-derived C-C chemokine that preferentially attracts naive T-cells. Nature.

[5] Hieshima K., Imai T., Baba M., Shoudai K., Ishizuka K., Nakagawa T., Tsuruta J., Takeya M., Sakaki Y., Takatsuki K., Miura R., Opdenakker G., van Damme J., Yoshie O., Nomiyama H. (1997). A novel human CC chemokine PARC that is most homologous to macrophage-inflammatory protein-1 alpha/LD78 alpha and chemotactic for T lymphocytes, but not for monocytes. J. Immunol..

[6] Lindhout E., Vissers J.L.M., Hartgers F.C., Huijbens R.J.F., Scharenborg N.M., figdor C.G., Adema G.J. (2001). The dendritic cell-specific CC-chemokine DC-CK1 is expressed by germinal center dendritic cells and attreact CD38-negative mantle zone B lymphocytes. J. Immunol..

[7] Reape T.J., Rayner K., Manning C.D., Gee A.N., Barnette M.S., Burnand K.G., Groot P.H. (1999). Expression and cellular localization of the CC chemokines PARC and ELC in human atherosclerotic plaques. Am. J. Pathol..

[8] Hägg D.A., Olson F.J., Kjelldahl J., Jernås M., Thelle D.S., Carlsson L.M.S., Fagerberg F., Svensson P.-A. (2009). Expression of Chemokine (C – C motif) ligand 18 in human macrophages and atherosclerotic plaques. Atherosclerosis.

[9] Kraajeveld A.O., de Jager S.C.A., de Jager W.J., Prakken B.J., McColl S.R., Haspels I., Putter H., van Berkel T.J.C., Nagelkerken L., Jukema J.W., Biessen E.A.L. (2007). CC Chemokine Ligand 5 (CCL5/RANTES) and CC Chemokine Ligand 18 (CCL18/PARC) are specific markers of refractory unstable angina pectoris and are transiently raised during severe ischemic symptoms. Circulation.

[10] de Jager S.C.A., Bongaerts B.W.C., Weber M., Kraaijeveld A.O., Rousch M., Dimmeler S., van Dieijen-Visser M.P., Cleutjens B.J.M., Nelemans P.J., van Berkel T.J.C., Biessen E.A.L. (2012). Chemokines CCL3/MIP1α, CCL5/RANTES and CCL18/PARC are independent risk predictors of short-term nortality in patients with acute coronary syndromes. PLoS One.

[11] Versteylen M.O., Manca M., Joosen I.A., Schmidt D.E., Das M., Hofstra I., Crijns H.J., Biessen E.A., Kietselaer B.L. (2016). CC chemokine ligands in patients presenting with stable chest pain: association with atherosclerosis and future cardiovascular events. Neth. Heart J..

[12] Pönitz V, Brügger-Andersen T, Pritchard D, Grundt H, Staines H, Nilsen DW; for the RACS study group. Activated Factor XII type A predicts long-term moratlity in patients admitted with chest pain, J Thromb Haemost. 2009 Feb; 7(2):277-87. Epub 2008 Dec 1. 10.1111/j.1538-7836.2008.03248.x 19054318

[13] Mjelva ØR, Pønitz V, Brügger-Andersen T, Grundt H, Staines H, Nilsen DW. Long-term prognostic utility of pentraxin 3 and D-dimer as compared to high-sensitivity C-reactive protein and B-type natriuretic peptide in suspected acute coronary syndrome. Eur J Prev Cardiol. 2016 Jul;23(11):1130-40. doi: 10.1177/2047487315619733. Epub 2015 Dec 3. 10.1177/2047487315619733 26635361

[14] León de la Fuente R, Naesgaard PA, Nilsen ST, Woie L, Aarsland T, Gallo P, Grundt H, Staines H, Nilsen DWT. B-type natriuretic peptide and high sensitive C-reactive protein predict 2-year All Cause Mortality in Chest Pain Patients: A Prospective Observational Study from Salta, Argentina. BMC Cardiovasc Disord 2011 Sep 29;11:57. 10.1186/1471-2261-11-57 PMC3199275 21958326

[15] Ruddox V., Mathisen M., Otterstad J.E. (2012). Prevalence and prognosis of non-specific chest pain among patients hospitalized for suspected acute coronary syndrome – a systematic literature search. BMC Med..

[16] Boot R.G., Verhoek M., de Fost M., Hollak C.E.M., Maas M., Bleijlevens B., van Bremen M.J., van Meurs M., Boven L.A., Laman J.D., Moran M.T., Cox T.M., Aerts J.M.F.G. (2004). Marked elevation of the chemokine CCL18/PARC in Gaucher disease: a novel surrogate marker for assessing therapeutic intervention. Blood.

[17] Bonaccio M., Di Castelnuovo A., Pounis G., De curtis A., Costanzo S., Persichillo M., Derletti C., Donati M.B., ce Gaetano G., Lacoviello L., Moli-sani Study Investigators (2016). A score of low-grade inflammation and risk of mortality: prospective findings from the Moli-sani study. Haematoligica.

[18] Danese E., Montagana M. (2016). An historical approach to the diagnostic biomarkers of acute coronary syndrome. Ann. Transl. Med..

