# Supplementary material for: Chemokine ligand 18 predicts all-cause mortality in patients hospitalized with chest pain of suspected coronary origin
Source: Int J Cardiol Cardiovasc Risk Prev. 2024 Mar 27;21:200264. doi: 10.1016/j.ijcrp.2024.200264 (PMC11002648; doi:10.1016/j.ijcrp.2024.200264)
Supplement: Multimedia component 1 [file mmc1.docx]

**Supplemental Figure 1.**

|  |  |  |  |  | | | | | | | |  |  |  |  |
| --- | --- | --- | --- | --- | --- | --- | --- | --- | --- | --- | --- | --- | --- | --- | --- |
|  |  |  |  | Total chest pain patients screened | | | | | | | |  |  |  |  |
|  |  |  |  | (*n* = 1211) | | | | | | | |  |  |  |  |
|  |  |  |  |  |  | |  |  | |  |  |  |  |  |  |
|  |  |  |  |  |  |  | |  | | Excluded because of non-coronary chest pain | | | |  |  |
|  |  |  |  |  |  |  | |  | | (*n* = 300) | | | |  |  |
|  |  |  |  |  |  |  | |  | |  |  |  |  |  |  |
|  |  |  |  |  |  |  | |  | | Other causes for exclusion^a^ | | | |  |  |
|  |  |  |  |  |  |  | |  | | (*n* = 40) | | | |  |  |
|  |  |  |  |  |  |  | |  | |  |  |  |  |  |  |
|  |  |  |  | Suspected coronary chest pain patients included in the RACS study | | | | | | | |  |  |  |  |
|  |  |  |  |  |  |  |  |  |  |  |  |  |  |  |  |
|  |  |  |  | (*n* = 871) | | | | | | | |  |  |  |  |
|  |  |  |  |  |  | |  |  |  | |  |  |  |  |  |
|  |  |  |  |  |  | |  |  |  | |  |  |  |  |  |
|  | STEMI | |  | NSTEMI | | |  |  | UAP | | |  | Non-ACS | |  |
|  | (*n* = 131 - 15.0%) | |  | (*n* = 255 - 29.3%) | | |  |  | (*n* = 82 - 9.4%) | | |  | (*n* = 403 -46.3%) | |  |
|  |  | |  |  | | |  |  |  | | |  |  | |  |

Abbreviations: RACS=”Risk Markers in the Acute Coronary Syndrome”. STEMI=ST-elevation myocardial infarction.

NSTEMI=Non-ST-elevation myocardial infarction. UAP=Unstable angina pectoris. Non-ACS=Non-acute coronary syndrome.

**Supplemental Figure 2.**


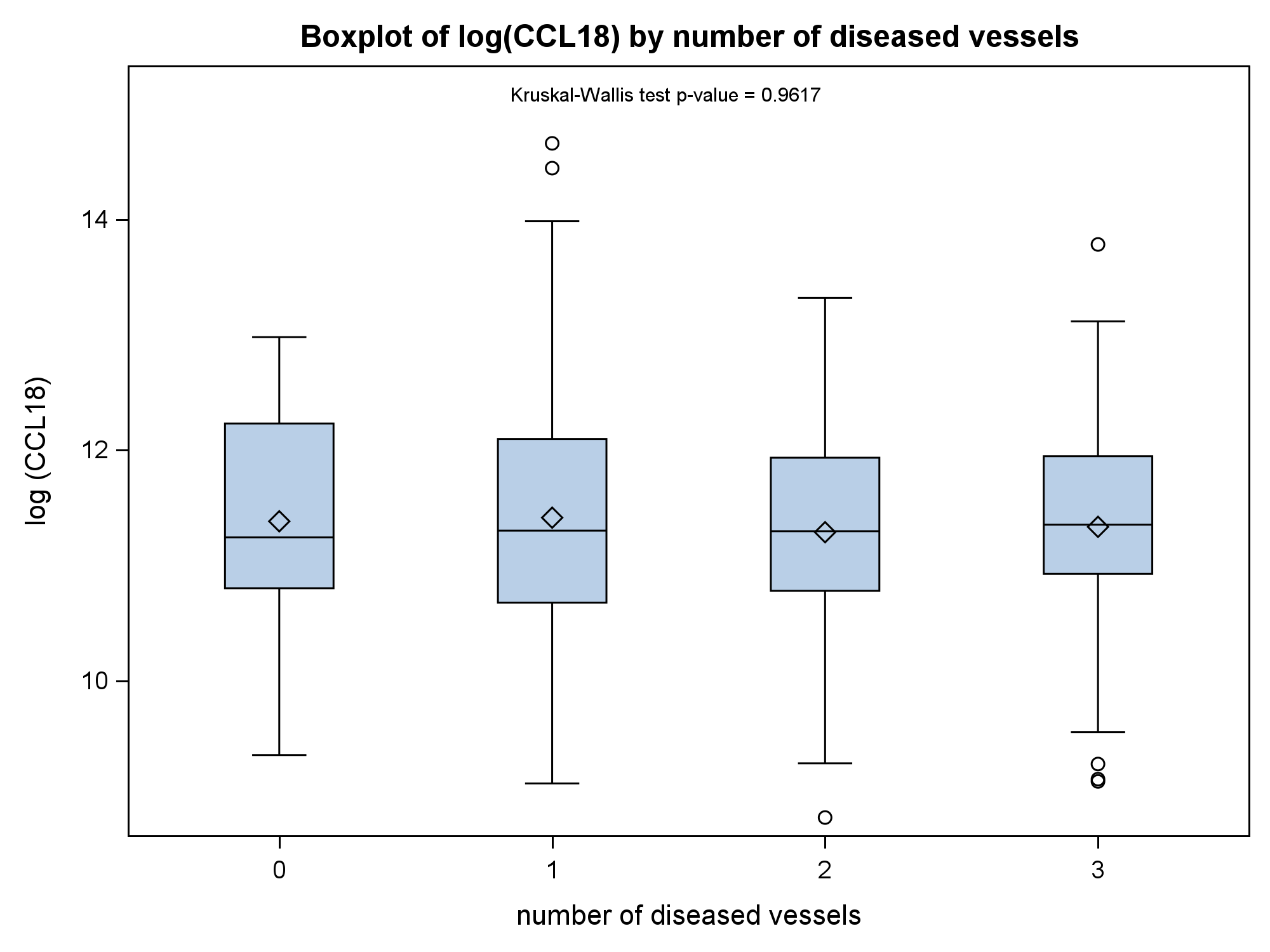


- The figure shows similar distributions of log_e_ CCL18

across the number of diseased vessels.

- There is no evidence of log_e_ CCL18 depending on the

number of diseased vessels (Kruskal-Wallis test p-value = 0.96).

**Supplemental Table 1.** Baseline characteristics in 386 patients with TnT > 50 ng/L, arranged in quartiles (Q) of CCL18, measured in arbitrary units (AU). Blood samples from 374 patients were available for measurement of CCL18.

|  |  |  |  |  | p_value | total |
| --- | --- | --- | --- | --- | --- | --- |
| \|  \| **Quartile1** \| **Quartile2** \| **Quartile3** \| **Quartile4** \| **p_value** \| **total** \| \| --- \| --- \| --- \| --- \| --- \| --- \| --- \| \|  \| **(n = 93)** \| **(n = 94)** \| **(n = 94)** \| **(n = 93)** \|  \| **(n = 374)** \| \| PARC (ngmL) \| 76.2 ( 63.8 - 88.7 ) \| 109.2 ( 102.0 - 119.3 ) \| 146.3 ( 137.9 - 157.6 ) \| 200.0 ( 177.9 - 244.8 ) \| <0.001 [2] \| 126.6 ( 95.3 - 165.8 ) \| \| Age (years) \| 67.2 ( 51.4 - 75.6 ) \| 72.6 ( 59.4 - 80.7 ) \| 75.3 ( 66.0 - 82.6 ) \| 74.9 ( 65.9 - 82.9 ) \| <0.001 [2] \| 72.7 ( 60.6 - 81.3 ) \| \| Male gender \| 59 ( 63.44 ) \| 58 ( 61.70 ) \| 68 ( 72.34 ) \| 69 ( 74.19 ) \| 0.168 [1] \| 254 ( 67.91 ) \| \| *Risk markers:* \|  \|  \|  \|  \|  \|  \| \| hs-CRP mg/L \| 3.3 ( 1.7 - 8.7 ) \| 4.1 ( 1.8 - 9.6 ) \| 6.3 ( 2.4 - 18.8 ) \| 9.4 ( 3.5 - 35.0 ) \| <0.001 [2] \| 5.0 ( 2.2 - 16.0 ) \| \| BNP pg/mL \| 77.0 ( 31.5 - 297.0 ) \| 95.5 ( 48.0 - 291.0 ) \| 216.0 ( 51.5 - 461.5 ) \| 224.0 ( 61.0 - 736.0 ) \| <0.001 [2] \| 137.0 ( 44.0 - 416.0 ) \| \| eGFR ml/min/1.73m2 \| 74.7 ( 58.6 - 88.1 ) \| 63.7 ( 48.6 - 77.4 ) \| 61.1 ( 47.1 - 71.1 ) \| 56.9 ( 37.5 - 71.5 ) \| <0.001 [2] \| 63.1 ( 48.0 - 76.7 ) \| \| Total chol. (mmol/L) \| 5.6 ( 4.9 - 6.2 ) \| 5.4 ( 4.7 - 6.4 ) \| 5.2 ( 4.2 - 5.8 ) \| 5.0 ( 4.0 - 6.0 ) \| 0.008 [2] \| 5.3 ( 4.5 - 6.1 ) \| \| TnT > 10 ng/L \| 93 ( 100.00 ) \| 94 ( 100.00 ) \| 94 ( 100.00 ) \| 93 ( 100.00 ) \|  \| 374 ( 100.00 ) \| \| *Smoking:* \|  \|  \|  \|  \| 0.047 [1] \|  \| \| Never smoked \| 26 ( 27.96 ) \| 30 ( 31.91 ) \| 33 ( 35.11 ) \| 33 ( 35.48 ) \|  \| 122 ( 32.62 ) \| \| Current smoker \| 38 ( 40.86 ) \| 29 ( 30.85 ) \| 34 ( 36.17 ) \| 18 ( 19.35 ) \|  \| 119 ( 31.82 ) \| \| Exsmoker \| 29 ( 31.18 ) \| 35 ( 37.23 ) \| 27 ( 28.72 ) \| 42 ( 45.16 ) \|  \| 133 ( 35.56 ) \| \| Hypertension \| 32 ( 34.41 ) \| 42 ( 44.68 ) \| 38 ( 40.43 ) \| 41 ( 44.09 ) \| 0.462 [1] \| 153 ( 40.91 ) \| \| Diabetes mell. type I \| 3 ( 3.23 ) \| 1 ( 1.06 ) \| 0 ( 0.00 ) \| 1 ( 1.08 ) \| 0.274 [1] \| 5 ( 1.34 ) \| \| Diabetes mell. type II \| 9 ( 9.68 ) \| 11 ( 11.70 ) \| 13 ( 13.83 ) \| 17 ( 18.28 ) \| 0.351 [1] \| 50 ( 13.37 ) \| \| Tot.chol. >6.5 mmol/L) \| 16 ( 17.20 ) \| 21 ( 22.34 ) \| 10 ( 10.64 ) \| 11 ( 11.83 ) \| 0.102 [1] \| 58 ( 15.51 ) \| \| *Hist. of heart disease:* \|  \|  \|  \|  \|  \|  \| \| Angina pectoris \| 29 ( 31.18 ) \| 40 ( 42.55 ) \| 44 ( 46.81 ) \| 43 ( 46.24 ) \| 0.109 [1] \| 156 ( 41.71 ) \| \| Myocardial infarction \| 21 ( 22.58 ) \| 30 ( 31.91 ) \| 27 ( 28.72 ) \| 39 ( 41.94 ) \| 0.037 [1] \| 117 ( 31.28 ) \| \| Previous CABG \| 7 ( 7.53 ) \| 9 ( 9.57 ) \| 7 ( 7.45 ) \| 6 ( 6.45 ) \| 0.879 [1] \| 29 ( 7.75 ) \| \| Previous PCI \| 7 ( 7.53 ) \| 4 ( 4.26 ) \| 9 ( 9.57 ) \| 8 ( 8.60 ) \| 0.537 [1] \| 28 ( 7.49 ) \| \| Heart failure \| 9 ( 9.68 ) \| 23 ( 24.47 ) \| 28 ( 29.79 ) \| 36 ( 38.71 ) \| <0.001 [1] \| 96 ( 25.67 ) \| \| *Prehospital treatment:* \|  \|  \|  \|  \|  \|  \| \| ACEI/ARB \| 21 ( 22.58 ) \| 31 ( 32.98 ) \| 30 ( 31.91 ) \| 34 ( 36.56 ) \| 0.201 [1] \| 116 ( 31.02 ) \| \| Beta-blocker \| 21 ( 22.58 ) \| 35 ( 37.23 ) \| 22 ( 23.40 ) \| 37 ( 39.78 ) \| 0.013 [1] \| 115 ( 30.75 ) \| \| Statins \| 25 ( 26.88 ) \| 30 ( 31.91 ) \| 28 ( 29.79 ) \| 26 ( 27.96 ) \| 0.882 [1] \| 109 ( 29.14 ) \| \| Aspirin \| 29 ( 31.18 ) \| 39 ( 41.49 ) \| 23 ( 24.47 ) \| 29 ( 31.18 ) \| 0.094 [1] \| 120 ( 32.09 ) \| |  |  |  |  |  | (n = 374) |
| Data are presented as median (interquartile range) or numbers (%). Abbreviations: hs-CRP, high-sensitivity C-reactive protein; BNP, B-type  natriuretic peptide; eGFR, estimated glomerular filtration rate; TnT, troponin-T; Total chol., total cholesterol; CABG, coronary artery bypass grafting; PCI, percutaneous coronary intervention. ACEI, Angiotensin-converting-enzyme inhibitor; ARB, Angiotensin receptor blocker. *For the diagnosis of an acute myocardial infarction, we applied a cut-off value for TnT of 50 ng/L and the lowest detectable value was 10 ng/L. [1] Chi-squared test. [2] Kruskal-Wallis test.  **Supplemental Table 2.** Baseline characteristics in 495 patients with TnT ≤ 50 ng/L, arranged in quartiles (Q) of CCL18, measured in arbitrary units (AU). Blood samples from 475 patients were available for measurement of CCL18. |  |  |  |  | 0.168 [1] | 254 ( 67.91 ) |
| \|  \| **Quartile1** \| **Quartile2** \| **Quartile3** \| **Quartile4** \| **p_value** \| **total** \| \| --- \| --- \| --- \| --- \| --- \| --- \| --- \| \|  \| **(n = 118)** \| **(n = 119)** \| **(n = 119)** \| **(n = 119)** \|  \| **(n = 475)** \| \| PARC (ngmL) \| 74.2 ( 63.3 - 81.7 ) \| 101.5 ( 95.7 - 109.9 ) \| 134.2 ( 123.4 - 145.4 ) \| 187.6 ( 166.6 - 211.1 ) \| <0.001 [2] \| 116.1 ( 89.5 - 154.2 ) \| \| Age (years) \| 63.0 ( 53.9 - 75.4 ) \| 69.6 ( 56.7 - 80.3 ) \| 73.0 ( 58.0 - 80.8 ) \| 77.8 ( 71.1 - 84.7 ) \| <0.001 [2] \| 72.3 ( 57.8 - 81.1 ) \| \| Male gender \| 67 ( 56.78 ) \| 63 ( 52.94 ) \| 70 ( 58.82 ) \| 66 ( 55.46 ) \| 0.830 [1] \| 266 ( 56.00 ) \| \| hs-CRP mg/L \| 2.4 ( 1.3 - 7.0 ) \| 2.9 ( 1.1 - 9.6 ) \| 3.5 ( 1.5 - 13.6 ) \| 5.0 ( 1.7 - 18.0 ) \| 0.012 [2] \| 3.3 ( 1.4 - 11.8 ) \| \| BNP pg/mL \| 52.0 ( 21.0 - 176.0 ) \| 58.0 ( 16.0 - 178.0 ) \| 81.5 ( 27.0 - 193.0 ) \| 179.0 ( 53.0 - 487.0 ) \| <0.001 [2] \| 80.0 ( 28.0 - 233.0 ) \| \| eGFR ml/min/1.73m2 \| 68.7 ( 58.7 - 80.8 ) \| 62.1 ( 50.1 - 73.2 ) \| 65.4 ( 50.2 - 75.9 ) \| 52.9 ( 38.2 - 68.2 ) \| <0.001 [2] \| 63.3 ( 49.3 - 74.9 ) \| \| Total chol. (mmol/L) \| 5.3 ( 4.4 - 6.3 ) \| 4.9 ( 4.2 - 5.9 ) \| 5.2 ( 4.2 - 6.2 ) \| 4.8 ( 3.9 - 5.6 ) \| 0.007 [2] \| 5.0 ( 4.2 - 5.9 ) \| \| TnT (> 10 ng/L) \| 10 ( 8.47 ) \| 19 ( 15.97 ) \| 15 ( 12.61 ) \| 40 ( 33.61 ) \| <0.001 [1] \| 84 ( 17.68 ) \| \| Smoking \|  \|  \|  \|  \| 0.278 [1] \|  \| \| Never smoked \| 41 ( 34.75 ) \| 49 ( 41.18 ) \| 47 ( 39.50 ) \| 59 ( 49.58 ) \|  \| 196 ( 41.26 ) \| \| Current smoker \| 32 ( 27.12 ) \| 22 ( 18.49 ) \| 25 ( 21.01 ) \| 21 ( 17.65 ) \|  \| 100 ( 21.05 ) \| \| Exsmoker \| 45 ( 38.14 ) \| 48 ( 40.34 ) \| 47 ( 39.50 ) \| 39 ( 32.77 ) \|  \| 179 ( 37.68 ) \| \| Hypertension \| 51 ( 43.22 ) \| 48 ( 40.34 ) \| 50 ( 42.02 ) \| 55 ( 46.22 ) \| 0.826 [1] \| 204 ( 42.95 ) \| \| Diabetes mellitus type I \| 2 ( 1.69 ) \| 0 ( 0.00 ) \| 0 ( 0.00 ) \| 1 ( 0.84 ) \| 0.293 [1] \| 3 ( 0.63 ) \| \| Diabetes mellitus type II \| 10 ( 8.47 ) \| 13 ( 10.92 ) \| 15 ( 12.61 ) \| 21 ( 17.65 ) \| 0.177 [1] \| 59 ( 12.42 ) \| \| Total chol. > 6.5 mmol/L \| 26 ( 22.03 ) \| 17 ( 14.29 ) \| 22 ( 18.49 ) \| 8 ( 6.72 ) \| 0.008 [1] \| 73 ( 15.37 ) \| \| *History of heart disease:* \|  \|  \|  \|  \|  \|  \| \| Angina pectoris \| 50 ( 42.37 ) \| 49 ( 41.18 ) \| 54 ( 45.38 ) \| 67 ( 56.30 ) \| 0.078 [1] \| 220 ( 46.32 ) \| \| Myocardial infarction \| 41 ( 34.75 ) \| 40 ( 33.61 ) \| 38 ( 31.93 ) \| 46 ( 38.66 ) \| 0.733 [1] \| 165 ( 34.74 ) \| \| Previous CABG \| 19 ( 16.10 ) \| 13 ( 10.92 ) \| 12 ( 10.08 ) \| 14 ( 11.76 ) \| 0.498 [1] \| 58 ( 12.21 ) \| \| Previous PCI \| 12 ( 10.17 ) \| 19 ( 15.97 ) \| 17 ( 14.29 ) \| 11 ( 9.24 ) \| 0.333 [1] \| 59 ( 12.42 ) \| \| Heart failure \| 25 ( 21.19 ) \| 22 ( 18.49 ) \| 30 ( 25.21 ) \| 55 ( 46.22 ) \| <0.001 [1] \| 132 ( 27.79 ) \| \| *Prehospital treatmen*t: \|  \|  \|  \|  \|  \|  \| \| ACEI/ARB \| 37 ( 31.36 ) \| 41 ( 34.45 ) \| 43 ( 36.13 ) \| 51 ( 42.86 ) \| 0.303 [1] \| 172 ( 36.21 ) \| \| Beta-blocker \| 46 ( 38.98 ) \| 49 ( 41.18 ) \| 42 ( 35.29 ) \| 54 ( 45.38 ) \| 0.451 [1] \| 191 ( 40.21 ) \| \| Statins \| 52 ( 44.07 ) \| 49 ( 41.18 ) \| 43 ( 36.13 ) \| 40 ( 33.61 ) \| 0.338 [1] \| 184 ( 38.74 ) \| \| Aspirin \| 53 ( 44.92 ) \| 54 ( 45.38 ) \| 51 ( 42.86 ) \| 44 ( 36.97 ) \| 0.536 [1] \| 202 ( 42.53 ) \| |  |  |  |  |  |  |
|  |  |  |  |  | <0.001 [2] | 5.0 ( 2.2 - 16.0 ) |

Data are presented as median (interquartile range) or numbers (%). Abbreviations: hs-CRP, high-sensitivity C-reactive protein; BNP, B-type

natriuretic peptide; eGFR, estimated glomerular filtration rate; TnT, troponin-T; Total chol., total cholesterol; CABG, coronary artery bypass grafting; PCI, percutaneous coronary intervention. ACEI, Angiotensin-converting-enzyme inhibitor; ARB, Angiotensin receptor blocker. *For the diagnosis of an acute myocardial infarction, we applied a cut-off value for TnT of 50 ng/L and the lowest detectable value was 10 ng/L. [1] Chi-squared test. [2] Kruskal-Wallis test

**Supplemental Table 3.**

Significant confounders for selected endpoints at 1, 2 and 7 years.

| A | Time (years) | Population | Table | Age/10 | BNPQ | TnT > .01 | Angina pectoris | CHF | Current smoking | MI | NIDDM | HEXSM | CABG |
| --- | --- | --- | --- | --- | --- | --- | --- | --- | --- | --- | --- | --- | --- |
| Death | 1 | all | 2A | 0,000 | 0,000 | 0,000 |  |  |  |  |  |  |  |
| Cardiac death | 1 | all | 2A | 0,000 | 0,002 | 0,000 |  |  |  |  |  |  |  |
| XXXXXXXXXXXXXXXXXXXXXXXX |  |  |  |  |  |  |  |  |  |  |  |  |  |
| Death | 2 | all | 2B | 0,000 | 0,000 | 0,003 |  |  |  |  |  |  |  |
| Cardiac death | 2 | all | 2B | 0,000 | 0,000 | 0,000 |  |  |  |  |  |  |  |
| XXXXXXXXXXXXXXXXXXXXXXXX |  |  |  |  |  |  |  |  |  |  |  |  |  |
| Death | 7 | all | 3A | 0,000 | 0,000 | 0,017 |  |  |  |  |  |  |  |
| Death or MI or stroke | 7 | all | 3B | 0,000 | 0,000 | 0,000 | 0,045 | 0,003 |  |  | 0,002 |  | 0,041 |
| XXXXXXXXXXXXXXXXXXXXXXXX |  |  |  |  |  |  |  |  |  |  |  |  |  |
| Cardiac death or MI or stroke | 2 | AMI | 4A |  | 0,007 |  |  | 0,001 | 0,004 |  |  |  |  |
| Cardiac death or MI or stroke | 2 | NON-AMI | 4A | 0,000 |  | 0,000 |  |  |  | 0,022 |  |  |  |
| XXXXXXXXXXXXXXXXXXXXXXXX |  |  |  |  |  |  |  |  |  |  |  |  |  |
| Death | 7 | AMI | 4B | 0,000 | 0,001 |  |  |  |  |  |  |  |  |
| Death or MI or stroke | 7 | AMI | 4B | 0,000 | 0,002 |  |  | 0,000 |  |  | 0,005 |  | 0,013 |
| XXXXXXXXXXXXXXXXXXXXXXXX |  |  |  |  |  |  |  |  |  |  |  |  |  |
| Death | 7 | NON-AMI | 4B | 0,000 |  | 0,000 |  | 0,046 | 0,031 |  |  |  |  |
| Death or MI or stroke | 7 | NON-AMI | 4B | 0,000 |  | 0,000 |  |  |  |  |  | 0,034 |  |

AMI=Acute Myocardial Infarction, Age/10=10 years interval, BNQ=Brain Natriuretic Peptide Quartile, TnT=Troponin T, CHF=Chronic Heart Failure, NIDDM=Non Insulin Diabetes Mellitus, HEXSM=Never & past smoking, CABG=Coronary Artery Bypass Grafting.

| **Supplemental Table 4. A & B.** Univariate and multivariable Cox regression model applying continuous log_e_-transformed values of baseline CCL18 values during 1 and 2 years follow-up, respectively, in 386 patients with an acute myocardial infarction (TnT > 0.05 ng/mL). (12 missing values in the univariate and age & gender adjusted and 25 missing values in the multivariable analysis).   \| **A.** \|  \| **All-cause mortality**  **1 year**  N=63 (16.8%)  M=61 (16.9%) \| \| \| **Cardiac death**  **1 year**  N=47 (12.6%)  M=46 (12.7%) \| \| \| **MI**  **1 year**  N=62 (16.6%)  M=61 (16.9%) \| \| \| **Stroke**  **1 year**  N=5 (1.3%)  M=4 (1.1%) \| \| \| \| --- \| --- \| --- \| --- \| --- \| --- \| --- \| --- \| --- \| --- \| --- \| --- \| --- \| --- \| \|  \|  \| **HR (95% CI)** \| **P-value** \| **HR (95% CI)** \| \| **P-value** \| **HR (95% CI)** \| \| **P-value** \| **HR (95% CI)** \| \| **P-value** \| \| **Univariate** \|  \| 1.22 (0.95 – 1.56) \| 0.12 \| 1.11 (0.83 – 1.49) \| \| 0.46 \| 1.10 (0.85 – 1.41) \| \| 0.47 \| 3.26 (1.33 – 7.99) \| \| 0.01 \| \| **Adjusted for gender** \|  \| 1.25 ( 0.97 - 1.60) \| 0.084 \| 1.15 ( 0.86 - 1.54) \| \| 0.34 \| 1.11 ( 0.86 - 1.43) \| \| 0.43 \| 3.20 ( 1.31 - 7.85) \| \| 0.011 \| \| **Adjusted for age and gender** \|  \| 0.98 ( 0.75 - 1.28) \| 0.87 \| 0.87 ( 0.64 - 1.19) \| \| 0.39 \| 0.97 ( 0.74 - 1.28) \| \| 0.85 \| 2.28 ( 0.91 - 5.73) \| \| 0.079 \| \| **Multivariable** \|  \| 0.94 (0.72 - 1.24) \| 0.68 \| 0.83 (0.60 - 1.13) \| \| 0.24 \| 0.89 (0.68 - 1.15) \| \| 0.36 \| 1.99 (0.80 – 4.99) \| \| 0.14 \|  \| **B.** \|  \| **All-cause mortality**  **2 years**  N=74 (19.8%)  M=71 (19.7%) \| \| \| **Cardiac death**  **2 years**  N=55 (14.7%)  M=53 (14.7%) \| \| \| **MI**  **2 years**  N=95 (25.4%)  M=93 (25.8%) \| \| \| **Stroke**  **2 years**  N=10 (2.7%)  M=9 (2.5%) \| \| \| \| \| --- \| --- \| --- \| --- \| --- \| --- \| --- \| --- \| --- \| --- \| --- \| --- \| --- \| --- \| --- \| \|  \|  \| **HR (95% CI)** \| **P-value** \| **HR (95% CI)** \| \| **P-value** \| **HR (95% CI)** \| \| **P-value** \| **HR (95% CI)** \| \| **P-value** \| \| **Univariate** \|  \| 1.27 (1.01 – 1.60) \| 0.042 \| 1.14 (0.87 – 1.49) \| \| 0.34 \| 1.14 (0.93 – 1.40) \| \| 0.21 \| 1.87 (1.00 – 3.50) \| \| 0.052 \| \| **Adjusted for gender** \|  \| 1.30 ( 1.03 - 1.64) \| 0.024 \| 1.18 ( 0.91 - 1.55) \| \| 0.21 \| 1.15 ( 0.94 - 1.42) \| \| 0.17 \| 1.83 ( 0.98 - 3.43) \| \| 0.059 \| \| **Adjusted for age and gender** \|  \| 1.02 ( 0.80 - 1.30) \| 0.89 \| 0.89 ( 0.67 - 1.19) \| \| 0.43 \| 0.96 ( 0.78 - 1.20) \| \| 0.74 \| 1.60 ( 0.82 - 3.11) \| \| 0.17 \| \| **Multivariable** \|  \| 0.99 (0.77 - 1.27) \| 0.96 \| 0.88 (0.63 - 1.21) \| \| 0.42 \| 0.85 (0.68 - 1.06) \| \| 0.15 \| Model did not converge \| \| \| \|   ___________________________________________________________________________________________________________________________  Abbreviations: N= Number in univariate analysis. M=Number in multivariable analysis. MI = Myocardial infarction. Stroke = Cerebral stroke. HR, Hazard Ratio; 95% CI, 95% confidence interval.  **Supplemental Table 5**. Univariate and multivariable Cox regression model applying continuous log_e_-transformed values of baseline CCL18 values  during 7 years follow-up in 386 patients with an acute myocardial infarction (TnT > 0.05 ng/mL), with 12 missing values in the univariate and age & gender adjusted and 25 missing values in the multivariable analysis.   \|  \|  \| **All-cause mortality**  **7 years**  N=150 (40.1%)  M=147 (40.7%) \| \| **MI**  **7 years**  N=117 (31.3%)  M=114 (31.6%) \| \| **Stroke**  **7 years**  N=19 (5.1%)  M=18 (5.0%) \| \| \| --- \| --- \| --- \| --- \| --- \| --- \| --- \| --- \| \|  \|  \| **HR (95% CI)** \| **P-value** \| **HR (95% CI)** \| **P-value** \| **HR (95% CI)** \| **P-value** \| \| **Univariate** \|  \| 1.42 (1.21 – 1.67) \| <0.001 \| 1.12 (0.93 – 1.34) \| 0.25 \| 1.13 (0.71 – 1.79) \| 0.62 \| \| **Adjusted for gender** \|  \| 1.44 ( 1.23 - 1.70) \| <0.001 \| 1.13 ( 0.94 - 1.37) \| 0.19 \| 1.13 ( 0.71 - 1.80) \| 0.60 \| \| **Adjusted for age and gender** \|  \| 1.11 ( 0.93 - 1.32) \| 0.23 \| 0.97 ( 0.80 - 1.18) \| 0.75 \| 0.93 ( 0.57 - 1.53) \| 0.79 \| \| **Multivariable** \|  \| 1.12 (0.93 - 1.33) \| 0.23 \| 0.89(0.72 – 1.09) \| 0.27 \| 0.92 (0.56 - 1.50) \| 0.73 \|  \|  \|  \| **All-cause mortality**  **or MI 7 years**  N=192 (51.3%)  M=188 (52.1%) \| \| **All-cause mortality**  **or MI or Stroke 7years**  N=199 (53.2%)  M=194 (53.7%) \| \| \| --- \| --- \| --- \| --- \| --- \| --- \| \|  \|  \| **HR (95% CI)** \| **P-value** \| **HR (95% CI)** \| **P-value** \| \| **Univariate** \|  \| 1.18 (1.02 – 1.37) \| 0.023 \| 1.19 (1.03 – 1.38) \| 0.017 \| \| **Adjusted for gender** \|  \| 1.20 ( 1.04 - 1.39) \| 0.013 \| \| 1.21 ( 1.05 - 1.39) \| 0.010 \| \| --- \| --- \| \| 0.01 \| \| **Adjusted for age and gender** \|  \| 0.99 ( 0.85 - 1.15) \| 0.88 \| 1.00 ( 0.86 - 1.16) \| 0.97 \| \| **Multivariable** \|  \| 0.94 (0.80 - 1.11) \| 0.47 \| 0.95 (0.81 - 1.11) \| 0.52 \|   ________________________________________________________________________________________________  Abbreviations: N= Number of events in the univariate analysis. M=Number of events in the multivariable analysis.  MI = Myocardial infarction. Stroke = Cerebral stroke. HR, Hazard Ratio; 95% CI, 95% confidence interval.  **Supplemental Table 6. A & B.** Univariate and multivariable Cox regression model applying continuous log_e_-transformed values of baseline CCL18 values during 1 and 2 years follow-up, respectively, in 485 patients with TnT ≤ 0.05 ng/mL. (10 missing values in the univariate and age & gender adjusted analysis and 26 missing values in the multivariable analysis).   \| **A.** \|  \| **All-cause mortality**  **1 year**  N=38 (8.0%)  M=37 (8.1%) \| **Cardiac death**  **1 year**  N=19 (4.0%)  M=18 (3.9%) \| **MI**  **1 year**  N=28 (5.9%)  M=26 (5.7%) \| **Stroke**  **1 year**  N=10 (2.1%)  M=10 (2.2%) \| \| --- \| --- \| --- \| --- \| --- \| --- \|  \|  \|  \| **HR (95% CI)** \| **P-value** \| **HR (95% CI)** \| \| \| **P-value** \| \| **HR (95% CI)** \| \| \| **P-value** \| \| **HR (95% CI)** \| \| **P-value** \| \| \| --- \| --- \| --- \| --- \| --- \| --- \| --- \| --- \| --- \| --- \| --- \| --- \| --- \| --- \| --- \| --- \| --- \| --- \| \| **Univariate** \|  \| 2.37 (1.67 – 3.35) \| <0.001 \| 2.26 (1.39 – 3.68) \| \| \| 0.001 \| \| 1.64 (1.11 – 2.42) \| \| \| 0.013 \| \| 1.48 (0.77 – 2.82) \| \| 0.24 \| \| \| **Adjusted for gender** \|  \| 2.31 ( 1.64 - 3.25) \| <0.001 \| \| 2.18 (1.36 - 3.50) \| 0.001 \| \| --- \| --- \| \| \| \| 0.001 \| \| 1.61 ( 1.10 - 2.37) \| \| \| 0.014 \| \| 1.46 ( 0.77 - 2.77) \| \| 0.25 \| \| \| **Adjusted for age and gender** \|  \| 1.69 ( 1.18 - 2.42) \| 0.004 \| 1.57 ( 0.96 - 2.57) \| \| \| 0.075 \| \| 1.25 ( 0.84 - 1.85) \| \| \| 0.27 \| \| 0.98 ( 0.51 - 1.90) \| \| 0.96 \| \| \| **Multivariable** \|  \| 1.51 (1.04 – 2.17) \| 0.029 \| 1.15 (0.68 - 1.94) \| \| \| 0.60 \| \| 0.92 (0.61 - 1.38) \| \| \| 0.67 \| \| 1.00 (0.52 – 1.92) \| \| 0.99 \| \| \| **B.** \|  \| **All-cause mortality**  **2 years**  N=61 (12.8%)  M=58 (12.6%) \| \| \| \| **Cardiac death**  **2 years**  N=29 (6.1%)  M=27 (5.9%) \| \| \| \| **MI**  **2 years**  N=56 (11.8%)  M=53 (11.5%) \| \| \| \| \| **Stroke**  **2years**  N=17 (3.6%)  M=17 (3.7%) \| \| \| \| \| \|  \|  \| **HR (95% CI)** \| **P-value** \| \| **HR (95% CI)** \| \| \| **P-value** \| \| \| **HR (95% CI)** \| \| **P-value** \| \| **HR (95% CI)** \| \| **P-value** \| \| \| **Univariate** \|  \| 1.97 (1.50 – 2.58) \| <0.001 \| \| 1.56 (1.06 – 2.28) \| \| \| 0.024 \| \| \| 1.45 (1.09 – 1.91) \| \| 0.009 \| \| 1.84 (1.11 – 3.06) \| \| 0.019 \| \| \| **Adjusted for gender** \|  \| 1.95 ( 1.49 - 2.55) \| <0.001 \| \| 1.55 ( 1.06 - 2.26) \| \| \| 0.024 \| \| \| 1.44 ( 1.09 - 1.91) \| \| 0.010 \| \| 1.82 ( 1.10 - 3.02) \| \| 0.019 \| \| \| **Adjusted for age and gender** \|  \| 1.39 ( 1.05 - 1.84) \| 0.020 \| \| 1.07 ( 0.72 - 1.58) \| \| \| 0.74 \| \| \| 1.11 ( 0.83 - 1.48) \| \| 0.47 \| \| 1.26 ( 0.75 - 2.12) \| \| 0.38 \| \| \| **Multivariable** \|  \| 1.12 (0.84 - 1.52) \| 0.42 \| \| Model did not converge \| \| \| \| \| \| 0.93 (0.7 - 1.23) \| \| 0.61 \| \| 1.33 (0.77 - 2.29) \| \| 0.31 \| \|   ___________________________________________________________________________________________________________________________  Abbreviations: N= Number in univariate analysis. M=Number in multivariable analysis. MI = Myocardial infarction. Stroke = Cerebral stroke. HR, Hazard Ratio; 95% CI, 95% confidence interval.  **Supplemental Table 7.** Multivariable Cox regression model applying continuous log_e_-transformed values of baseline CCL18 values during 1, 2, 3, 4, 5, 6, 7 years follow-up, respectively, in 871 patients with a suspected acute coronary syndrome. (51 missing values in the multivariable analysis). |  |  |  |  | <0.001 [2] | 137.0 ( 44.0 - 416.0 ) |
| --- | --- | --- | --- | --- | --- | --- | --- | --- | --- | --- | --- | --- | --- | --- | --- | --- | --- | --- | --- | --- | --- | --- | --- | --- | --- | --- | --- | --- | --- | --- | --- | --- | --- | --- | --- | --- | --- | --- | --- | --- | --- | --- | --- | --- | --- | --- | --- | --- | --- | --- | --- | --- | --- | --- | --- | --- | --- | --- | --- | --- | --- | --- | --- | --- | --- | --- | --- | --- | --- | --- | --- | --- | --- | --- | --- | --- | --- | --- | --- | --- | --- | --- | --- | --- | --- | --- | --- | --- | --- | --- | --- | --- | --- | --- | --- | --- | --- | --- | --- | --- | --- | --- | --- | --- | --- | --- | --- | --- | --- | --- | --- | --- | --- | --- | --- | --- | --- | --- | --- | --- | --- | --- | --- | --- | --- | --- | --- | --- | --- | --- | --- | --- | --- | --- | --- | --- | --- | --- | --- | --- | --- | --- | --- | --- | --- | --- | --- | --- | --- | --- | --- | --- | --- | --- | --- | --- | --- | --- | --- | --- | --- | --- | --- | --- | --- | --- | --- | --- | --- | --- | --- | --- | --- | --- | --- | --- | --- | --- | --- | --- | --- | --- | --- | --- | --- | --- | --- | --- | --- | --- | --- | --- | --- | --- | --- | --- | --- | --- | --- | --- | --- | --- | --- | --- | --- | --- | --- | --- | --- | --- | --- | --- | --- | --- | --- | --- | --- | --- | --- | --- | --- | --- | --- | --- | --- | --- | --- | --- | --- | --- | --- | --- | --- | --- | --- | --- | --- | --- | --- | --- | --- | --- | --- | --- | --- | --- | --- | --- | --- | --- | --- | --- | --- | --- | --- | --- | --- | --- | --- | --- | --- | --- | --- | --- | --- | --- | --- | --- | --- | --- | --- | --- | --- | --- | --- | --- | --- | --- | --- | --- | --- | --- | --- | --- | --- | --- | --- | --- | --- | --- | --- | --- | --- | --- | --- | --- | --- | --- | --- | --- | --- | --- | --- | --- | --- | --- | --- | --- | --- | --- | --- | --- | --- | --- | --- | --- | --- | --- | --- | --- | --- | --- | --- | --- | --- | --- | --- | --- | --- | --- | --- | --- | --- | --- | --- | --- | --- | --- | --- | --- | --- | --- | --- | --- | --- | --- | --- | --- | --- | --- | --- | --- | --- | --- | --- | --- | --- | --- | --- | --- | --- | --- | --- | --- | --- | --- | --- | --- | --- | --- | --- | --- | --- | --- | --- | --- | --- | --- | --- | --- | --- | --- | --- | --- | --- | --- | --- | --- | --- | --- | --- | --- | --- | --- | --- | --- | --- | --- | --- | --- | --- | --- | --- | --- | --- | --- | --- | --- | --- | --- | --- | --- | --- | --- | --- | --- | --- | --- | --- | --- | --- | --- | --- | --- | --- | --- | --- | --- | --- | --- | --- | --- | --- | --- | --- | --- | --- | --- | --- | --- | --- | --- | --- | --- | --- | --- | --- | --- | --- | --- | --- | --- | --- | --- | --- | --- | --- | --- | --- | --- | --- | --- | --- | --- | --- |

|  | **Cardiac death or MI or stroke** | |  | **All-cause mortality** | |
| --- | --- | --- | --- | --- | --- |
|  | **HR (95% CI)** | **p-value** |  | **HR (95% CI)** | **p-value** |
| 1 year | 0.94 (0.78 – 1.12) | 0.46 |  | 1.06 (0.86 – 1.30) | 0.60 |
| 2 years | 0.92 (0.79 – 1.06) | 0.23 |  | 1.06 (0.88 - 1.27) | 0.55 |
| 3 years |  |  |  | 1.18 (1.00 - 1.39) | 0.054 |
| 4 years |  |  |  | 1.17 (1.01 – 1.36) | 0.041 |
| 5 years |  |  |  | 1.17 (1.02 - 1.34) | 0.028 |
| 6 years  7 years |  |  |  | 1.13 (1.00 - 1.29)  1.14 (1.01 – 1.29) | 0.059  0.030 |
|  |  |  |  |  |  |

**Supplemental Table 8**. Univariate and multivariable Cox regression model applying continuous log_e_-transformed values of baseline CCL18 values

during 7 years follow-up in 485 patients with with TnT ≤ 0.05 ng/mL. (10 missing values in the univariate and adjusted for age & gender, 26 missing values in the multivariable analysis).

|  |  | **All-cause mortality**  **7 years**  N=177 (37.3%)  M=170 (37.0%) | | | **MI**  **7 years**  N=82 (17.3%)  M=79 (17.2%) | | **Stroke**  **7 years**  N=33 (6.9%)  M=33 (7.2%) | |
| --- | --- | --- | --- | --- | --- | --- | --- | --- |
|  |  | | **HR (95% CI)** | **P-value** | **HR (95% CI)** | **P-value** | **HR (95% CI)** | **P-value** |
| **Univariate** |  | 1.77 (1.51 – 2.08) | | <0.001 | 1.44 (1.14 – 1.81) | 0.002 | 1.31 (0.91 – 1.88) | 0.14 |
| **Adjusted for gender** |  | 1.77 ( 1.51 - 2.08) | | <0.001 | 1.44 ( 1.14 - 1.82) | 0.002 | 1.31 ( 0.91 - 1.88) | 0.14 |
| **Adjusted for age and gender** |  | 1.29 ( 1.09 - 1.52) | | 0.003 | 1.10 ( 0.86 - 1.40) | 0.46 | 0.96 ( 0.66 - 1.38) | 0.82 |
| **Multivariable** |  | 1.20 (1.02 - 1.42) | | 0.030 | 0.98 (0.77 – 1.25) | 0.87 | 0.95 (0.66 - 1.37) | 0.77 |

|  |  | **All-cause mortality**  **or MI 7 years**  N=205 (43.2%)  M=197 (42.9%) | | **All-cause mortality**  **or MI or Stroke 7years**  N=214 (45.1%)  M=206 (44.9%) | |
| --- | --- | --- | --- | --- | --- |
|  |  | **HR (95% CI)** | **P-value** | **HR (95% CI)** | **P-value** |
| **Univariate** |  | 1.60 (1.38 – 1.86) | <0.001 | 1.56 (1.35 – 1.80) | <0.001 |
| **Adjusted for gender** |  | 1.60 ( 1.38 - 1.86) | <0.001 | 1.56 ( 1.35 - 1.80) | <0.001 |
| **Adjusted for age and gender** |  | 1.20 ( 1.03 - 1.40) | 0.017 | 1.17 ( 1.01 - 1.35) | 0.041 |
| **Multivariable** |  | 1.11 (0.95 - 1.30) | 0.19 | 1.09 (0.93 - 1.26) | 0.28 |

________________________________________________________________________________________________

Abbreviations: N= Number of events in the univariate analysis. M=Number of events in the multivariable analysis.

MI = Myocardial infarction. Stroke = Cerebral stroke. HR, Hazard Ratio; 95% CI, 95% confidence interval.

**Supplemental Table 9.**

Patients in the non-AMI population (TnT ≤ 50 ng/L) with non-missing CCL18 measurements (n = 475).

| Index diagnosis | CCL18 Quartile based on both AMI and non-AMI patients | | | | |
| --- | --- | --- | --- | --- | --- |
|  | Quartile1 | Quartile2 | Quartile3 | Quartile4 | Total |
| MI | 1 | 1 | 0 | 2 | 4 |
| UAP | 12 | 17 | 19 | 33 | 81 |
| NON-ACS | 118 | 106 | 100 | 66 | 390 |
| Total | 131 | 124 | 119 | 101 | 475 |

Ignoring the four patients with an MI diagnosis, no significant interaction effect was observed between

index diagnosis and CCL18 quartile (p=0.44).
